# The Anesthetic Complexity of Eisenmenger Syndrome: A Clinical Case

**DOI:** 10.7759/cureus.54285

**Published:** 2024-02-16

**Authors:** Rúben Calaia, Neuza Machado, Juliana Branquinho, Eduarda Figueiredo, Carla Pereira, Alexandra Guedes

**Affiliations:** 1 Anesthesiology, Unidade Local de Saúde Viseu Dão-Lafões, Viseu, PRT

**Keywords:** down syndrome, monitored anesthetic care, ketamine, dexmedetomidine, eisenmenger syndrome

## Abstract

Eisenmenger syndrome (ES) is a complex, multisystemic, and rare clinical entity, given that currently, most congenital heart diseases can be corrected in childhood. The high anesthetic risk in these patients poses a challenge for anesthesiology. There are few cases described in the literature of anesthetic approaches using ketamine and dexmedetomidine in ES cases, particularly under Monitored Anesthesia Care (MAC). We describe the clinical case of a 40-year-old patient with trisomy 21, intellectual disability, and ES secondary to a single atrioventricular (AV) valve, scheduled for cranial magnetic resonance imaging (MRI) under sedation due to a suspected space-occupying lesion. Sedation was performed under MAC with dexmedetomidine and ketamine. The procedure proceeded without complications. The anesthetic approach in ES patients, given the clinical complexity, requires planning by a multidisciplinary team and should be tailored to the procedure and its duration.

## Introduction

Eisenmenger syndrome (ES) is a clinical entity with multiorgan involvement due to the presence of cardiac anomalies that may occur at the level of the interventricular septum (IVS), interatrial, atrioventricular (AV), or patent ductus arteriosus associated with a left-to-right shunt. The persistence of these defects leads to progressive increases in pressures on the pulmonary circulation in the medium and long term, resulting in remodeling of the microvasculature and obstruction of blood flow. These factors contribute to increased pressures in the pulmonary circulation and the subsequent development of a right-to-left shunt. ES is a rare condition, occurring in about 3% of patients with mild defects in the IVS and about 50% of patients with major defects in the IVS (>1.5 cm) [1]. The clinical signs resulting from this syndrome are related to multiorgan involvement, such as hypoxemia, central cyanosis, pulmonary hypertension, bleeding diathesis, and hemoptysis [1]. The prognosis for ES patients remains unfavorable, with a 10-year survival rate of about 57% [1]. The anesthetic approach in these patients becomes challenging not only due to the heterogeneity/severity of clinical presentation and the profile of each patient but also due to the high risk of cardiovascular complications, thromboembolic events, arrhythmias, and death [2]. Therefore, any anesthetic procedure should be carefully planned and discussed with a multidisciplinary team to minimize morbidity and mortality. This clinical case describes an anesthetic approach with a level of sedation suitable for magnetic resonance imaging (MRI) in a patient with trisomy 21 and ES undergoing diagnostic imaging.

## Case presentation

**Figure 1:**
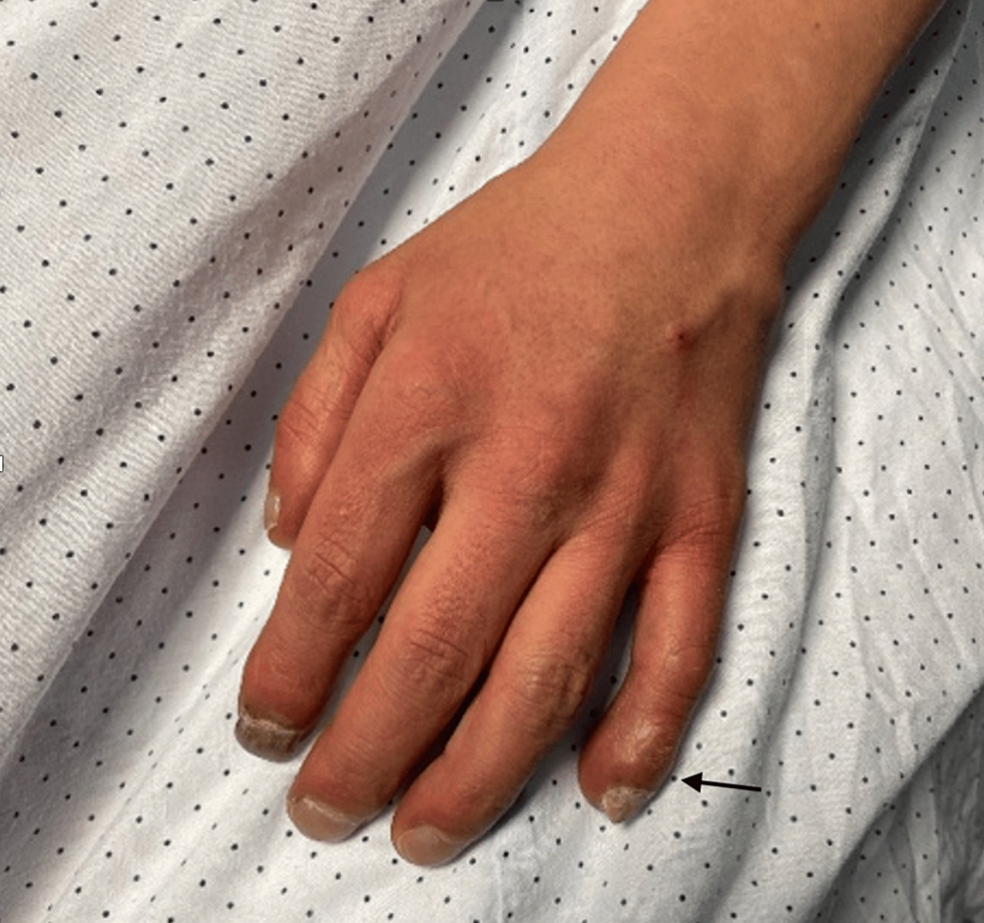
Clubbing (arrow)

We describe the clinical case of a 40-year-old patient, classified as American Society of Anesthesiologists, with Physical Status IV due to trisomy 21 associated with intellectual disability and ES developed due to congenital heart disease related to atrioventricular septal defect with a single AV valve and secondary polycythemia with a hematocrit of 60.2% due to cardiopulmonary condition. The patient was admitted to the neurosurgery department for a cranial MRI due to neurological deficits and generalized tonic-clonic seizures. Given the patient's clinical context, collaboration with anesthesiology was requested for an MRI under sedation for a differential diagnosis of abscess vs. neoplasm. During the pre-anesthetic evaluation, the patient presented with central cyanosis and peripheral oxygen saturation in ambient air between 85% and 90%, without signs of respiratory distress. On physical examination, the patient had clubbing (Figure 1). A transthoracic echocardiogram was requested, confirming the presence of a single AV valve (Video 1) with a mild central insufficiency jet, ventricular ejection fraction of 60.2%, and dilation of the pulmonary artery trunk. Analytically, the patient presented polycythemia and thrombocytopenia (Table 1).

**Video 1:** Transthoracic echocardiogram

**Table 1:** Laboratory investigation

Laboratory investigation	Laboratory value	Reference range
Leukocytes	19.70 x 10^9^/L	4.50–11.50 x 10^9^/L
Erythrocytes	6.50 x 10^12^/L	4.00–5.40 x 10^12^/L
Haemoglobin	21.6 g/dL	14.0–18.0 g/dL
Haematocrit	60.2%	40–54%
Platelet count	81,0000/μL	150,000–450,000/μL

**Figure 2:**
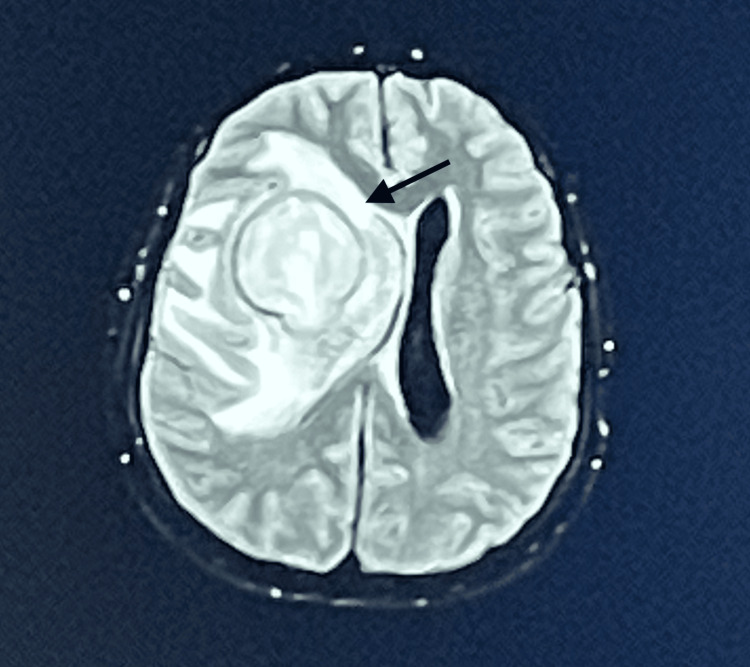
Right fronto-insular glioma (arrow)

The proposed anesthetic procedure was explained to the patient's family, and due to the patient's emotional dependence on the family member, their presence was requested during the MRI. Before the start of the procedure, standard ASA monitoring was performed, and a nasal oxygen cannula was placed with a flow of 2 L/min adapted to a capnograph (Smart CapnoLine® Plus O2). Subsequently, sedation was performed under monitored anesthesia care (MAC) with an initial bolus of 10 mg of intravenous ketamine, followed by a continuous infusion of dexmedetomidine at 0.5 µg/kg/h + ketamine at 0.4 mg/kg/h. The procedure lasted approximately 20 minutes with the maintenance of ventilatory and hemodynamic stability and patient immobility. Baseline peripheral oxygen saturations (85-90%) were maintained, and capnography allowed observation of an adequate respiratory rate (average of 20 breaths per minute). After the MRI, the patient was admitted to the post-anesthetic care unit for surveillance and monitoring and then readmitted to the neurosurgery ward. The definitive diagnosis was compatible with a right fronto-insular glioma (Figure 2). Due to the high anesthetic and surgical risks, palliative treatment was chosen, focusing on epileptogenic activity and comfort measures. One week after the MRI, the patient was admitted to the emergency room in cardiac arrest and was declared dead following the event.

## Discussion

Regardless of the approach and procedure to be performed, the anesthetic approach for patients with ES presents a significant challenge. Thus, the anesthetic focus essentially lies in preventing the worsening of the right-to-left shunt by controlling pulmonary vascular resistance but also in reducing oxygen consumption and preventing arrhythmogenic phenomena. Therefore, the choice of general anesthesia under mechanical ventilation, positive pressure, and positive end-expiratory pressure (PEEP) can increase the pressure on the right side, and this can worsen the right-to-left shunt and worsen hypoxemia [3], which is why we chose to perform MAC. Furthermore, MAC with dexmedetomidine is an approach to managing these patients, especially when they present other co-morbidities, such as cognitive deficits [4]. The titration of dexmedetomidine and ketamine is essential to ensuring hemodynamic balance and sedation suitable for MRI. Both drugs allow for conscious sedation through the maintenance of airway reflexes and spontaneous ventilation [5]. On the one hand, dexmedetomidine is a highly selective α2 agonist with anxiolytic, sedative, and analgesic properties and has the potential to prevent increased intracranial pressure [6,7]. At the nervous system level, it has a sympatholytic effect that hemodynamically produces a biphasic response when administered as a bolus followed by continuous infusion: initially, with bolus administration, there is an increase in peripheral vascular resistance and blood pressure, leading to reflex bradycardia; subsequently, with continuous infusion, the opposite occurs, i.e., a decrease in peripheral vascular resistance and blood pressure [7]. For this reason, instead of administering a bolus, a continuous infusion at 0.5 µg/kg/h was initiated to avoid hemodynamic variation in the initial phase. On the other hand, ketamine is a sympathomimetic that opposes the sympatholytic effect of dexmedetomidine, and its administration immediately before the start of the dexmedetomidine infusion is essential. The pharmacological combination of these two sedative/hypnotic agents ensures the maintenance of hemodynamic stability without an increase in peripheral vascular resistance, and the tendency to cause bradycardia from dexmedetomidine is balanced by the tendency to produce tachycardia from ketamine [5,7]. The antisialagogue effect of dexmedetomidine counterbalances the sialorrheic effect of ketamine, preventing the occurrence of laryngospasm and consequent airway compromise during the procedure [5,8]. Additionally, dexmedetomidine has a significant contribution to the pulmonary circulation. According to recent studies, this drug has an anti-inflammatory effect by inhibiting the cell proliferation of smooth muscle cells in the endothelium of the pulmonary circulation [9]. The cardioprotective effect that dexmedetomidine has demonstrated may be an advantage in approaching patients with cardiac pathology [10]. Finally, the occurrence of delirium after the procedure associated with ketamine administration is reduced with the concomitant use of dexmedetomidine, which, in this case, is an important factor [4]. Despite the importance of the adopted anesthetic approach, emotional support is a crucial factor in the success of this approach, especially in this case, and we considered the presence of a family member during the procedure to be fundamental.

## Conclusions

This clinical case demonstrated that the use of ketamine and dexmedetomidine could be a safe and effective alternative for sedation in patients with severe congenital heart diseases, allowing, under MAC, the performance of complementary diagnostic means where patient immobility is essential, along with sedation that does not compromise ventilation and airway defense reflexes. The simultaneous use of these two drugs allowed for balancing the cardio-stimulatory effects of ketamine with the sympatholytic effects of dexmedetomidine at the administered dose, ensuring an adequate hemodynamic response and spontaneous ventilation. The use of transesophageal echocardiography has proven to be an asset in these cases as it allows real-time monitoring of intracardiac shunt and cardiac output; however, in our institution, this resource is not available.
